# Emergent Properties of Giant Vesicles Formed by a Polymerization-Induced Self-Assembly (PISA) Reaction

**DOI:** 10.1038/srep41534

**Published:** 2017-01-27

**Authors:** Anders N. Albertsen, Jan K. Szymański, Juan Pérez-Mercader

**Affiliations:** 1 Department of Earth and Planetary Sciences and Origins of Life Initiative, Harvard University, Cambridge, Massachusetts 02138, United States; 2 Santa Fe Institute, Santa Fe, New Mexico 87501, United States

## Abstract

Giant micrometer sized vesicles are of obvious interest to the natural sciences as well as engineering, having potential application in fields ranging from drug delivery to synthetic biology. Their formation often requires elaborate experimental techniques and attempts to obtain giant vesicles from chemical media in a one-pot fashion have so far led to much smaller nanoscale structures. Here we show that a tailored medium undergoing controlled radical polymerization is capable of forming giant polymer vesicles. Using a protocol which allows for an aqueous reaction under mild conditions, we observe the macroscale consequences of amphiphilic polymer synthesis and the resulting molecular self-assembly using fluorescence microscopy. The polymerization process is photoinitiated by blue light granting complete control of the reaction, including on the microscope stage. The self-assembly process leads to giant vesicles with radii larger than 10 microns, exhibiting several emergent properties, including periodic growth and collapse as well as phototaxis.

Vesicles are ubiquitous in biology: they form the cornerstone of biological compartmentalization. Complex semipermeable vesicles with diameters over 5 μm provide living systems with a means to help generate and maintain the free-energy gradients necessary for Life^1^. Natural vesicle membranes are made of phospholipids, amphiphilic molecules which are replenished and synthesized during complex metabolic processes. In recent years, a large amount of effort has been devoted to studying vesicles made of artificial amphiphilic block copolymers known as polymersomes^2^,^3^,^4^,^5^. Just like phospholipids of biological origin, these synthetic polymers are composed of two or more blocks differing by hydrophobicity. Replacing lipids with polymers leads to improved vesicle resiliency and opens up diverse functionalization opportunities, allowing for introduction of functional groups responsive to stimuli such as light, temperature or pH^6^,^7^,^8^. Additionally, it opens new avenues for mimicking life and investigating some of the processes behind the origin of life.

Although the chemical synthesis of amphiphile molecules leading to polymersomes is far simpler than for phospholipids, the exact pathway of their formation in different environments^9^,^10^,^11^ is not yet fully understood. However, notable progress has taken place in recent years^12^,^13^,^14^,^15^,^16^. There are now chemical methods under the acronym PISA (Polymerization Induced Self-Assembly) capable of generating from simpler components solutions of self-assembled polymer structures (including worms, spheres or vesicles^17^) at relatively high solids concentrations (10% by weight and up), something not typically achieved with techniques such as solvent replacement, where the vesicle solutions are much more dilute.

A typical PISA experiment starts out as a monomer solution in a suitable solvent such as water, a simple alcohol^18^ or a hydrocarbon^19^. This is followed by the polymerization process, in most cases the versatile RAFT (Reversible Addition-Fragmentation-chain Transfer), which produces the amphiphiles that will gradually self-assemble into structures within the same reaction vessel. It is a “one pot reaction”. However, the worms, spheres and vesicles are typically 10’s to 100’s of nanometers in size, and current PISA does not give access to giant vesicles (diameter > 1 μm) which are relevant in many areas, including synthetic biology, and whose evolution is more easily studied and documented using optical and fluorescence microscopy^20^.

Recent preliminary work conducted in our group^21^ demonstrated the possibility of observing large-scale phase separation in a polymerization medium directly with fluorescence microscopy. Multitude of structures resulted, including phase-separated droplets, polymer aggregates and vesicles attached to larger structures. Here, we expand upon this result in order to focus exclusively on freely moving vesicles and their properties. In contrast to previous work, for example by Oana and co-workers^22^ who demonstrated growth of giant vesicles from optically trapped droplets of a polyion complex and that the thus formed vesicles reverted to droplet form by switching the laser beam off, we show here how by using a photocontrolled and carefully tailored version of PISA an initially homogeneous polymerization medium produces phase-separated droplets and sustains *in situ* light-induced growth of giant vesicles (approximately 10 to 15 μm in diameter). This differentiates our system from a phospholipid-based photocontrolled medium reported recently, featuring only static vesicles^23^. Photoinitiated PISA processes have been explored in the literature^24^,^25^, but, as mentioned above, the vesicles formed are in the nanometer size range and are not known to display either internal activity or dynamical cooperative behaviors.

The vesicles thus generated will eventually burst if the controlling illumination stays on, but can be made stable for extended periods of time through illumination followed by dark periods. We also report on the existence of cyclic vesicle formation, growth and collapse processes providing a pathway for a form of simplified polymerization-induced replication which displays the hallmarks of selective competition.

## Results

### Chemistry/synthesis

**Figure 1:**
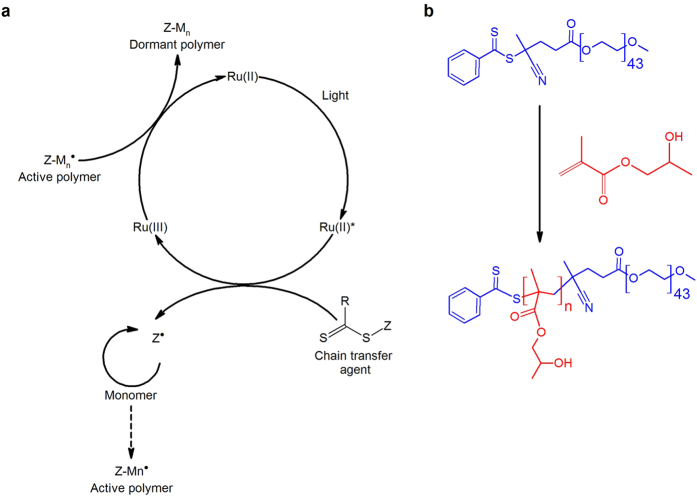
(**a**) Kinetic scheme of the light-induced processes taking place during biphasic polymerization of 2-hydroxypropyl methacrylate, adapted from Xu *et al*., *Macromolecules*, **2014**, *47*, 4217–4229. (**b**) Reaction scheme for the RAFT polymerization of 2-hydroxypropyl methacrylate controlled by a PEG-functional RAFT chain transfer agent as studied in the present work.

**Figure 2:**
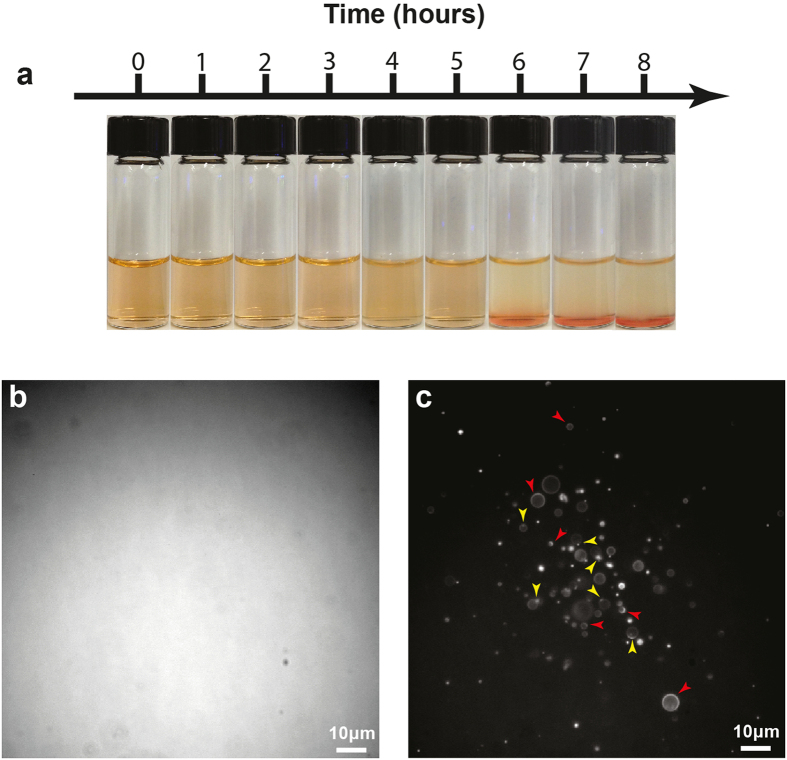
(**a**) Collage showing the reaction mixture throughout the PISA polymerization process, left to right. (**b**) shows the solution initially after 8 hours of blue light illumination in the vial and (**c**) shows the solution after 3 hours of blue light illumination on the microscope. Note the size of the polymersomes in c, the sample was stained with 4 μM rhodamine 6 G to record the micrographs. The yellow arrowheads in c point to monomer droplets enclosed in the membrane of the vesicles, the red arrowheads point to polymersomes where the monomer is spread over a larger portion of the membrane.

The polymerization medium we investigate is a reversible addition-fragmentation-chain transfer (RAFT)^26^ polymerization system featuring a water-soluble monomer, 2-hydroxypropyl methacrylate (HPMA), typically used in PISA formulations. We make two important modifications to the system. First, we introduce photocontrol, exploiting a ruthenium(II) based photosensitizer whose ability to initiate free radical polymerization is well-known^27^,^28^ (Fig. 1). Second, we modify the length of the poly(ethylene glycol) (PEG)-based RAFT chain transfer agent (CTA) so that undesirable nanosized structures are less likely to form. As the polymerization progresses the HPMA polymer becomes increasingly hydrophobic, but this PEG chain nevertheless ensures that the polymerized product will be amphiphilic (Fig. 2).

Having the reaction initiated by a photochemical process has several advantages. The ruthenium complex supplies a continuous and constant amount of radicals under a given light intensity. It has two well-defined absorbance bands, one in the UV range and one centered at 450 nm^29^, while remaining transparent to a range of wavelengths across the visual spectrum. The latter part of the spectrum can be used to study the morphologies and the dynamics of the polymersomes by using fluorescent dyes which associate with the vesicles while minimizing photobleaching. Another useful property of the ruthenium complex is that it is fluorescent when illuminated at 450 nm and it emits at around 600 nm. Using this signal it is possible to map the microscopic distribution of the ruthenium complex in the sample over time. As radicals are only supplied when the sample is illuminated with the correct wavelength, complete external control of the HPMA polymerization is achieved. Having this degree of control enables the study of morphologies at different stages of the PISA process since the polymerization can be terminated and reinitiated at will.

For a weight ratio of HPMA equal to 10% (target degree of polymerization (DP) = 200), the reaction medium underwent phase separation and a polymer phase with density exceeding that of the bulk aqueous medium precipitated out of the aqueous solution. Phase separation in these conditions was described in detail in our previous publication^21^. For lower degrees of polymerization, the required ratio of monomer to CTA was achieved through a further decrease in monomer concentration. Interestingly, no macroscale phase separation was observed for samples with target DP equal to 100 or less. All of these mixtures exhibited slight turbidity, and DLS measurements on a representative DP = 65 sample revealed structures in a broad size distribution of 30–3000 nm with a maximum at 600 nm (see the S.I. Figure 1). Proton NMR was used to measure the degree of polymerization of the samples, S.I. Figure 2. The spectra prove that the polymerization is relatively slow as conversion does not typically exceed 30%. The polymer molecular weight is consequently below the target and therefore a considerable amount of monomer still is present in the system, which allows for subsequent continued polymerization.

The samples for which the target DP was 65 and 75 (HPMA concentrations 3.25% and 3.75% w/w, respectively) showed reproducible formation of giant vesicles from an initially macro- and microscopically homogenous solution. We therefore focused on these formulations and investigated these phenomena and the behavior of the formed vesicles in greater detail.

### Giant vesicles

**Figure 3:**
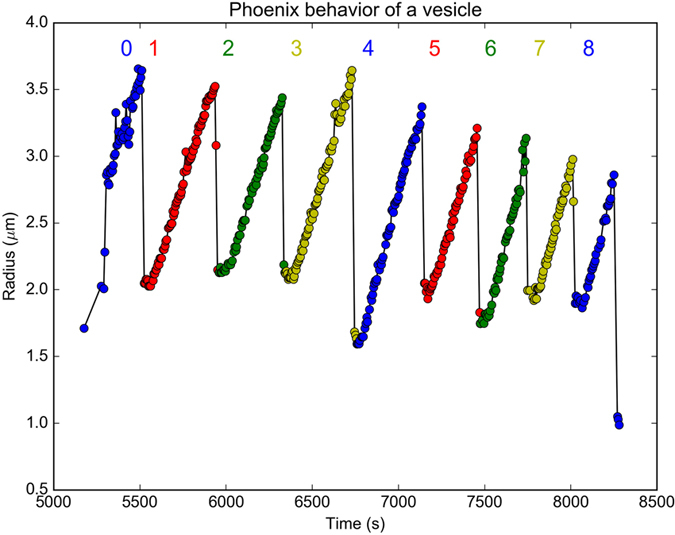
Trace of several polymersomes volume (sharing a common progenitor) over time showing the cyclic growth and collapse behavior when exposed to blue light. With each successive cycle the maximal size of the polymersome is decreasing. The different polymersomes were tracked and their area was measured using a computer algorithm. The oscillations span around 3000 seconds.

**Figure 4:**
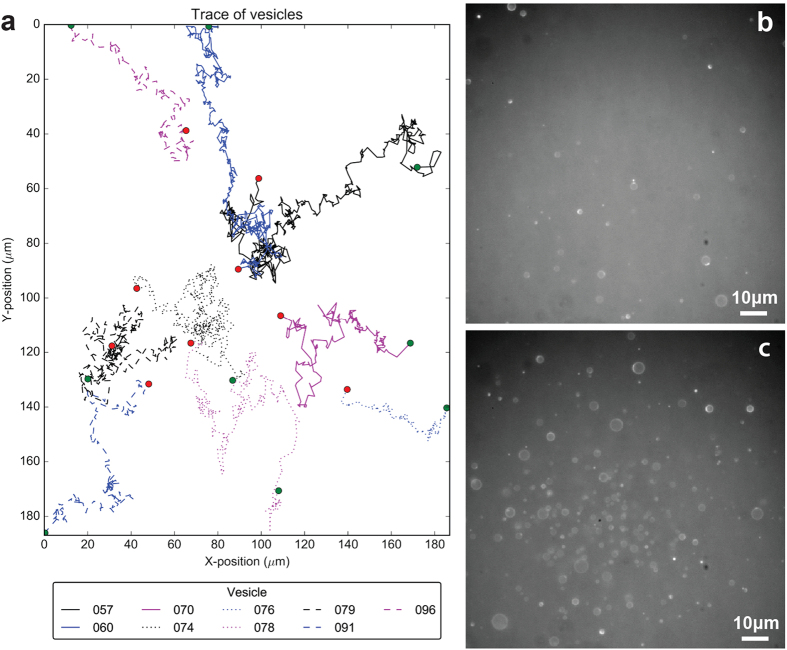
(**a**) Trace of the movement of a set of vesicles displaying chemotaxis. The collective movement of the vesicle population results in their concentration near the center of the frame where the light is the most intense. (**b**) Micrograph of the solution at the beginning of the experiment and (**c**) a micrograph at the end of the experiment. The computer trace was generated from a series of micrographs collected during a single experiment.

The PISA reaction used here forms both amphiphiles and vesicles in an aqueous “one-pot” reaction. The polymer self-assembly process occurs over several hours starting from a homogeneous solution, Fig. 2a, and results in significant changes at both the macroscopic, Fig. 2a, and microscopic levels, Fig. 2b and c. The vesicles formed by this process are polydisperse, see S.I. Figure 3, the largest are around 10 μm in diameter which is 10 to 100 times larger than previously reported for the PISA process. This qualifies the vesicles to be categorized as “giant”, if the size categorization for liposomes is applied^20^. In addition, the larger vesicles approach the size of living cells^1^. This, along with their long term stability, see S.I. Figure 4, enables traditional living cell microscopy methods to be applied to investigate the system. It also implies that these vesicles are subject to the same physical effects and constraints as their biological counterparts. The membranes of the vesicles contained structures exclusive to this environment. These structures were monomer inclusions in the form of droplets, Fig. 2c yellow arrows, and smears, Fig. 2c red arrows.

Since the system was stable over extended periods of time it was possible to microscopically study the effects of prolonged exposure to blue light (which activates the RAFT process), as well as other emergent properties and dynamics not present immediately after the polymersome formation.

### Phoenix behavior

Once giant polymersomes have formed their emergent properties become apparent. Most striking is the cyclic growth and collapse of the vesicles when exposed to constant blue light (450 nm).

**Figure 5:**
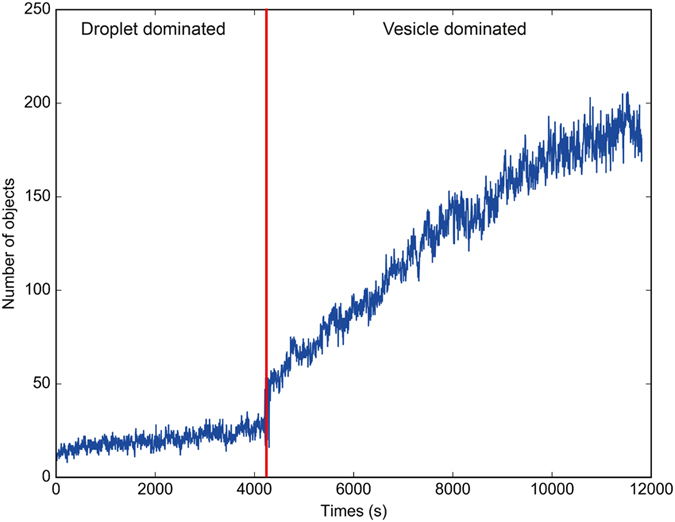
The number of objects, vesicles and droplets, over time as recorded by a computer algorithm. The droplet to vesicle phase transition visualized by the red line, highlighting a dramatic increase in the number of objects in each recorded microscope frame at the onset of vesicle formation.

During these cycles a nascent vesicle starts as a droplet of material which begins to grow as a vesicle, and continues until it reaches a maximum sustainable size as seen in SI Movie 1. At this critical size, the membrane ruptures and the structure collapses back into the initial droplet state from which it had emerged. The structures remain in this state until the growth starts anew and ends in yet another collapse. The individual structures undergo this cyclic “phoenix” behavior several times, see Fig. 3, SI Movie 1 and S.I. Figure 5.

The maximum size the vesicles can accommodate decreases slightly over the course of several cycles, Fig. 3, indicating that material is lost to the solution during the collapse phase. During the cycles monomer is consumed resulting in a dramatic decrease in the number of droplet inclusions and smears in the vesicle membrane over time. As the system matures the number of vesicles increases, the size distribution is skewed towards larger structures while also increasing the fraction of smaller structures, see S.I. Figure 3.

That the blue light induces the creation of the phoenix cycles becomes clear as this vesicle growth rate decreases considerably when the blue light intensity is reduced, see S.I. Figure 6. In complete absence of blue light, the polymersomes still undergo growth and collapse cycles although at a much slower rate, see the SI material 6. (Note that without blue light both the polymerization and the PISA process cannot occur and therefore, in these conditions, the growth of the vesicles must necessarily have a different origin.)

### Phenomenology of Phoenix vesicles and their population

As a first approximation, the radius of the phoenix vesicles can be used as a proxy for vesicle size under the assumption of the vesicles being perfect spheres. Its time evolution carries information on vesicle formation mechanism and the causes for their individual behavior as members of a population. The statistical study of this quantity as a function of time reveals that in each phoenix cycle the data is best fitted by a logistic curve^30^ (the underlying RAFT polymerization reaction is also known to follow sigmoidal kinetics^17^), a typical phenomenological indicator of autocatalytic or self-organized growth, which points to the existence of various distinct phases during vesicle growth. Specifically, during the initial phases we see a slow growth in size later followed by an exponentially accelerated phase and, finally, we see a decrease in the growth rate after a substantial local monomer depletion has taken place. This affects both the membrane and the internal vesicle bulk properties (cf. SI Movie 1). The membrane surface tension and the osmotic imbalance between the interior and the environment of the vesicle are therefore simultaneously affected and controlled by the details of the polymerization process. Together they lead to a scenario where the driving force behind the growth in vesicle size and subsequent vesicle collapse can be described in terms of an osmotic imbalance and the resulting transport of water from the outside medium into the polymersomes^31^.

Indeed, the polymerization of the hydrophobic chains (increasing the D.P. lowers the surface tension of the vesicle wall, which reduces the osmotic stress on the membrane) enables the vesicle to grow until a critical size is reached. The influx of water swells the polymersomes until the PISA process is no longer able to supply enough amphiphile polymer to counteract the stress on the membrane, at which point the membrane ruptures and the vesicle collapses into a droplet. Osmotic effects have been shown by Chen *et al*.^32^ to be able to promote growth of oleate vesicles and eventually induce competition for resources, which can be thought of as the initial steps of competition and evolutionary selection.

In other words, as osmotic pressure inside the vesicle falls (due to monomer depletion) the membrane acquires defects and becomes increasingly porous and locally weakened and, finally, the external pressure due to the influx of water into the vesicles forces their collapse into a smaller (because of chemical depletion due to polymerization) droplet than the original one from which the first phoenix generation of the vesicle (the “incarnation”) sprouted. Finally, the phoenix process cannot go on because the amount of monomer is not sufficient to feed the PISA process.

### Phototaxis and self-organized population growth via a dynamical phase transition

While at the phoenix stage, a moving and growing vesicle population is established, as can be seen in SI Movie 1. Using automated computer analysis, the individual vesicles were identified and their movements traced. The collected traces, Fig. 4a, confirm the observations from the micrograph time series, Fig. 4b and c. The data clearly shows that the polymersomes converge near the center of the frame and the concentration of vesicles in that area increases. In the absence of blue light, the polymersomes move in a non-directional and random manner, with computer analysis of the movements of the population of vesicles not revealing any discernible patterns or preferred direction of motion, see S.I. Figure 7. This proves that the directional movement is light dependent and suggests a primitive form of phototaxis as in simpler organisms.

The mechanism for the phototaxis can be understood in terms of a Marangoni instability forming around the membrane of the self-assembled polymersomes. (Directional movement due to a Marangoni instability has been described previously by Hanczyc *et al*., Toyota *et al*., and others^33^,^34^,^35^,^36^). In our case the Marangoni instability arises from the PISA process responding to the blue light gradient: since the polymerization process is more efficient in the region of vesicle surface that faces the most intense blue light, this area becomes more amphiphilic. The increase in amphiphillicity, in turn, decreases the surface tension of the membrane in the aqueous solution and creates a surface tension gradient over the membrane. This gradient induces a Marangoni effect which generates movement of water from the region of low surface tension, where the light is the most intense, to the region of higher surface tension, where the light is the least intense. Thus the moving water provides a force field that directs the polymersomes towards the area with the highest light intensity.

We observe that while our PISA system evolves from a droplet-dominated to a vesicle-dominated population, a significant change takes place in the ratio of the number of vesicles to that of droplets seen in the system, Fig. 5. Indeed, during the droplet phase the HPMA monomer is the main structural component into which the growing amphiphiles eventually condense. During this phase only a small number of structures can be accommodated, both because the monomer is quite water soluble (13% w/v^17^) and because of the poor surfactant qualities of the polymer (due to a low average degree of polymerization S.I. Figure 2). The stability of the droplets depends on a surfactant and these must therefore require a large number of polymer chains to remain stable, significantly reducing the amount available for other aggregates, such as micelles and worms. Dynamic light scattering data acquired before the onset of droplet formation does not suggest a significant presence of aggregates smaller than 100 nm, see S.I. Figure 1. Consequently, in this initial phase the system exhibits only a slow linear growth in the number of structures. However, after roughly 4000 s when the RAFT chemistry goes through the exponential growth phase in the D.P., the system undergoes a sharp transition into a vesicle-dominated phase and the number of (phoenix) vesicles in the system grows non-linearly. The increase in the D.P. allows the amphiphiles to form structures independently of the monomer concentration, and the number of structures that can be accommodated by the system automatically becomes amphiphile limited, as the amphiphiles are now the main structure-forming component. The combination of a gradient, such as the one provided by phototaxis, and the non-linearity present in the evolution of the D.P. then leads to the induction of the dynamical phase transition seen in Fig. 5^37^.

## Discussion

By using optical monitoring techniques, combined with computer vision and automated analysis of a light-controlled PISA process calculated to yield polymersomes of several microns in diameter in an aqueous medium, we discover the existence of a self-organized regime of consecutive polymer vesicle growth and collapse with a net increase in the polymersome population of the system. The cyclic episodes, which typically last for 10 minutes, can be explained in terms of osmotic pressure effects coupled with the consumption of the monomeric substrates contained in the droplet into which the synthesized nanometer scale amphiphilic diblock copolymers initially self-assemble. The observed net oscillatory vesicle population grows in a manner that reminds one of some elementary modes of sustainable (while there is available “food”!) population growth seen among living systems. The data supports an interpretation in terms of a micron scale self-assembled molecular system capable of embodying and mimicking some aspects of “simple” extant life, including self-assembly from a homogenous but active chemical medium, membrane formation, metabolism, a primitive form of self-replication, and hints of elementary system selection due to a spontaneous light triggered Marangoni instability.

## Methods

The chemicals for the experiments were purchased from either Sigma Aldrich or Alfa Aesar and used without further purification.

### Preparation of the standard polymerization induced self-assembly (PISA) samples

13.9 mg (6.95 μmol) of the RAFT agent (Poly(ethylene glycol) methyl ether 4-cyano-4-(phenylcarbonothioylthio)pentanoate, Sigma) was weighed in a vial. To the solid 63.44 μL (0.45 mmol) of the liquid monomer (hydroxypropyl methacrylate, mixture of isomers, 98%, stab. with ca 0.02% 4-methoxyphenol, Alfa Aesar) was added, to obtain a polymer target degree of polymerization of 65; for a target D.P. of 75 73.13 μL (0.52 mmol) was added to the solid RAFT agent. The mixture of 1996.74 μL of water (Chromasolv Plus for HPLC, Sigma) and 3.26 μL of 8.5 mM Ru(bpy)_3_^2+^ (Ruthenium tris 2,2′-bipyridyl chloride, Sigma) was added. The mixture was vortexed yielding a transparent, slightly orange-pink solution.

#### Initiation method 1

To initiate the polymerization, the sample was illuminated with LED lights in a spectroscopic cuvette thermostated at 30 °C, without stirring, for 16 hours, or in a vial with stirring for 8 hours.

#### Initiation method 2

Alternatively, the samples remained in the mixing vial, a stir bar was added and the vial was placed in a custom-made illumination chamber for 8 h with stirring.

### Microscopy

As the next step, small aliquots of the partially polymerized mixtures were transferred to a frame sealed, 65 μL (Frame-Seal™ Incubation Chambers, BioRadCA, USA), microscope slide and observed using fluorescence microscopy after staining with the fluorescent dye Rhodamine 6 G for better image quality. The fully automated fluorescence microscope setup allowed for a degree of control over illumination intensity and wavelength, and we exploited this to significantly limit the photobleaching of the dye and investigate effects of different illumination conditions and over long periods of illumination. We used cycles of 5 seconds of blue light illumination to promote the polymerization followed by 20 milliseconds of green light to read out the morphology of the sample through the Rhodamine 6 G distribution. The microscope observations were conducted on a Zeiss microscope (Axio Observer A1) equipped with a fluorescence light source (X-Cite 120Q) and filter setup (BD Carv II). The filters we used were for blue light excitation λ = 455–495 nm and for green light excitation λ = 540–590 nm both with an emission window of λ = 600–650 nm. The microscope setup was controlled using μManager software^38^.

### NMR sample preparation and measurements

After removing aliquots for fluorescence microscopy, the remainder of the sample was transferred to a 1.5 mL microcentrifuge tube and freeze-dried. The solids remaining in the tube were then dissolved in an NMR solvent. The proton NMR spectra were acquired in dimethyl sulfoxide-d_6_ or methanol-d_4_ at 25 °C on a 500 MHz Varian Unity/Inova spectrometer.

### Generating the size trace

The frames recorded by the microscope during the investigation of the growth and division were analyzed by a computer algorithm that located any structures in the frame, that being either oil droplets or vesicles. The centroid of the individual structures as well as their area was found and recorded using an edge filter.

The resulting datasets were used as the basis for further analysis using Python scripts. These scripts were used to monitor the size of the vesicles during their growth and division cycles, the resulting data sets were used to obtain the best fit of the data in order to derive the laws governing the growth of the structures. Additional scripts were written to track the path of individual vesicles through the data set. This would help determine if the vesicles were subject to Brownian motion or were moving in a specific direction. A script was written to measure the size of all structures in each frame in order to generate the size distribution histograms and their fits for each individual frame.

## Additional Information

**How to cite this article**: Albertsen, A. N. *et al*. Emergent Properties of Giant Vesicles Formed by a Polymerization-Induced Self-Assembly (PISA) Reaction. *Sci. Rep.* **7**, 41534; doi: 10.1038/srep41534 (2017).

**Publisher's note:** Springer Nature remains neutral with regard to jurisdictional claims in published maps and institutional affiliations.

## Supplementary Material

Supplementary Information

Supplementary Movie 1
