# Supplementary material for: Emergent Properties of Giant Vesicles Formed by a Polymerization-Induced Self-Assembly (PISA) Reaction
Source: Sci Rep. 2017 Jan 27;7:41534. doi: 10.1038/srep41534 (PMC5270245; doi:10.1038/srep41534)
Supplement: Supplementary Information [file srep41534-s1.pdf]

Supporting Information

for

EMERGENT PROPERTIES OF GIANT VESICLES FORMED BY A POLYMERIZATION-INDUCED SELF-ASSEMBLY (PISA) REACTION

by

A. N. Albertsen, J. K. Szymański, and J. Pérez-Mercader

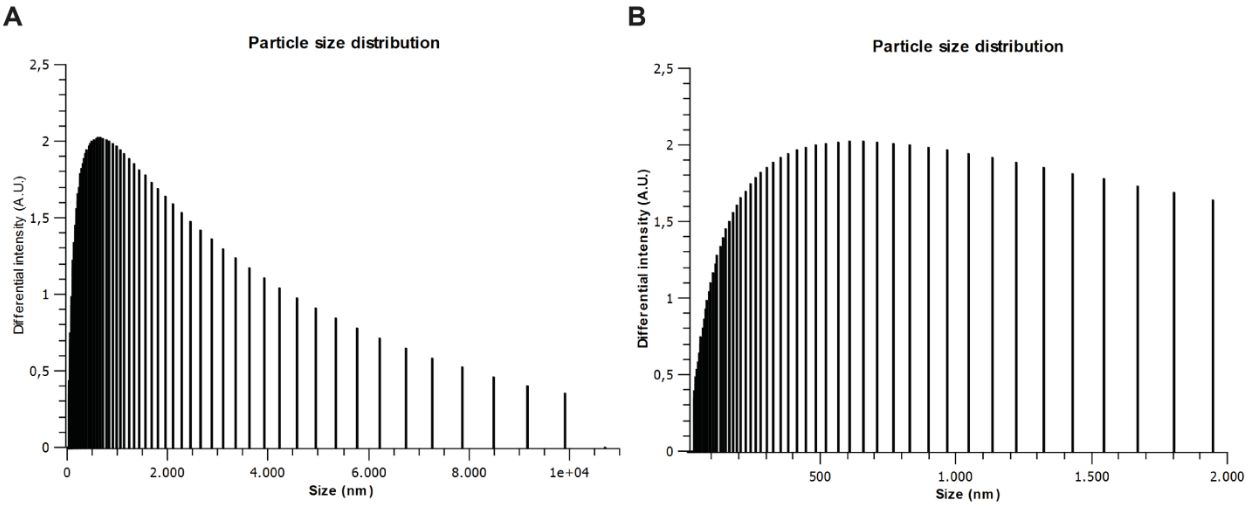

Figure S1: A) DLS data showing the size distribution of the structures formed in the reaction solution after 16 hours of blue light illumination. **B)** Detailed view of the data below 2 µm. The data was collected before the onset of vesiculation while the sample was macro- and microscopically homogeneous. This corresponds well to a maximum differential intensity which indicates a particle size of 608 nm.

The size distribution of the PISA reaction mixture was measured using DLS before the onset of vesicle formation in order to determine the size of the prevesicular aggregates, Supporting Figure 1. The scattering data revealed that less than 12% of the prevesicular aggregates were smaller than 100 nm, which rules out a significant presence of micelles in the solution.

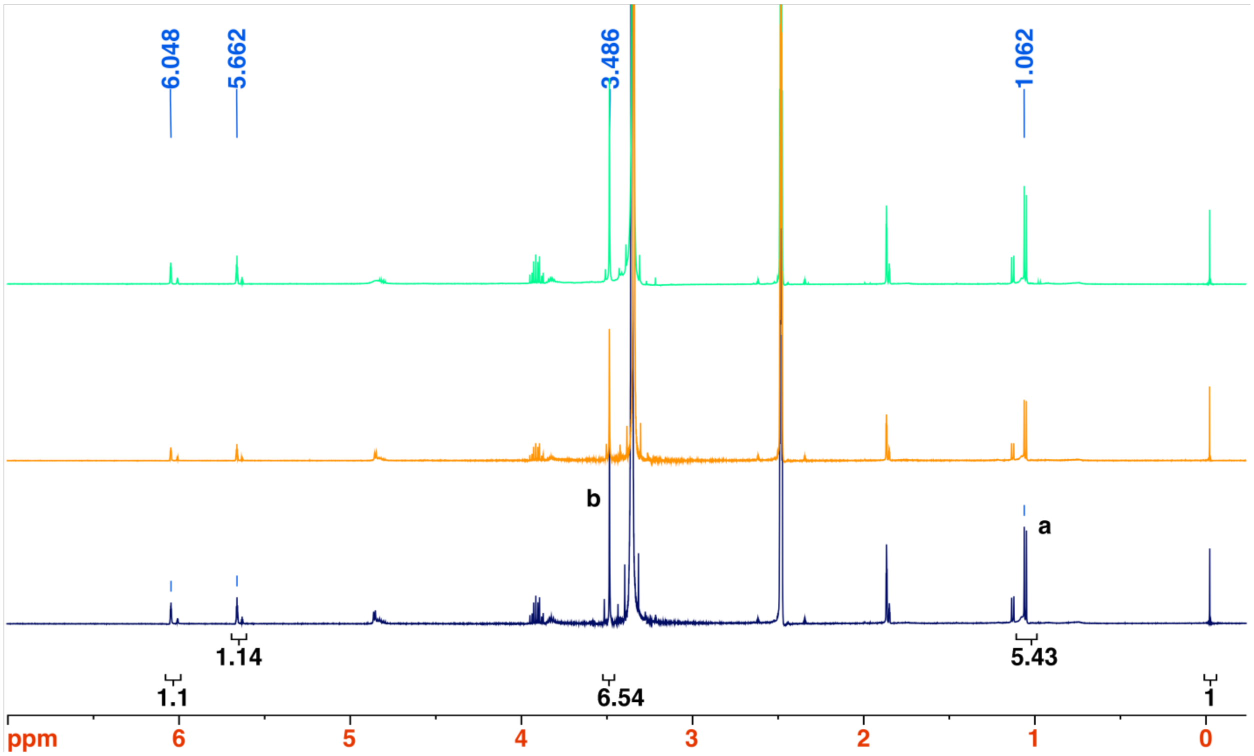

Figure S2. ^{1}H NMR spectra of freeze-dried material from three different poly(ethylene glycol)-*block*-poly(2-hydroxypropylmethacrylate)(PEG-PHPMA) solutions shown to form vesicles. Peaks labeled **a**atδ = 1.062 ppm correspond to terminal methyl groups in the side chains of the PHPMA block; peaks labeled bat δ = 3.486 ppm correspond to protons in the main PEG chain. The peaks in the region δ = 5.5 – 6.5 ppm are vinyl protons from unreacted monomer. Conversion and degree of polymerization were calculated from these integral values. As an example, the sample whose spectrum is given in dark blue is characterized by 39.2 % conversion and D.P. of 19 (theoretical D.P. for target of 65 at this conversion equals 25).

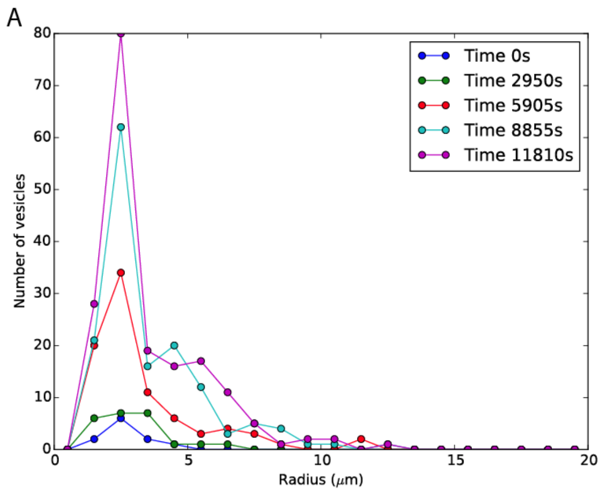

Figure S3: Evolution of the size distribution of the tracked objects from before the onset of vesicle formation until the stabilization in the number of "phoenix" vesicles. Note the increase in 5 μm objects after the onset of vesicle formation.

Supporting Figure 3 shows the evolution of the size distribution of the sample over time, as traced by the computer algorithm. As the population evolves and the individual vesicles undergo successive phoenix cycles, the size distribution of the population changes as well. Over time a shoulder to the main peak at 2.5 μm gains a shoulder centered around 6 μm and a tail at 12 μm. The size distribution is based on the micrographs recorded during an experiment. All vesicles detected by this method are larger than what previously has been reported in the literature [1-10].

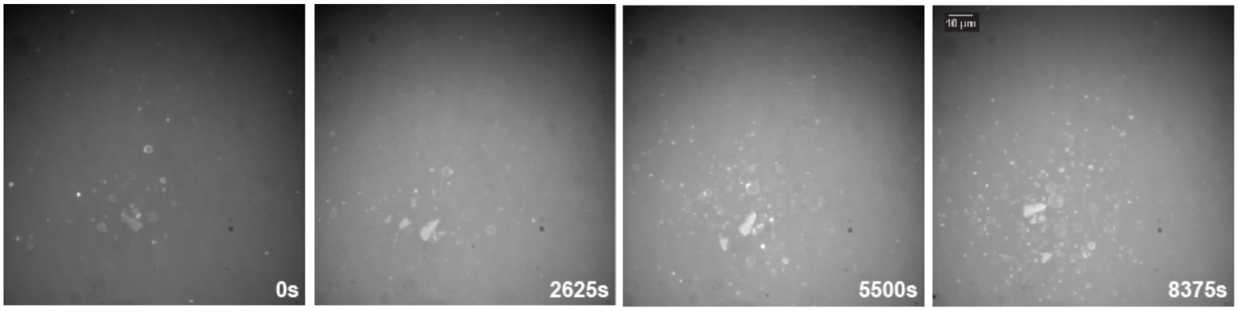

Figure S4: Time lapse of a vesicle population showing the evolution and stability of the polymersomes in the absence of blue light. The scale bar is 10 μm and applies to all frames. The sample was stained with rhodamine 6G.

The polymersome population was stable over several hours, Supporting Figure 4, which enabled the investigation of the system over longer periods of time. This time series covered more than 2.5 hours.

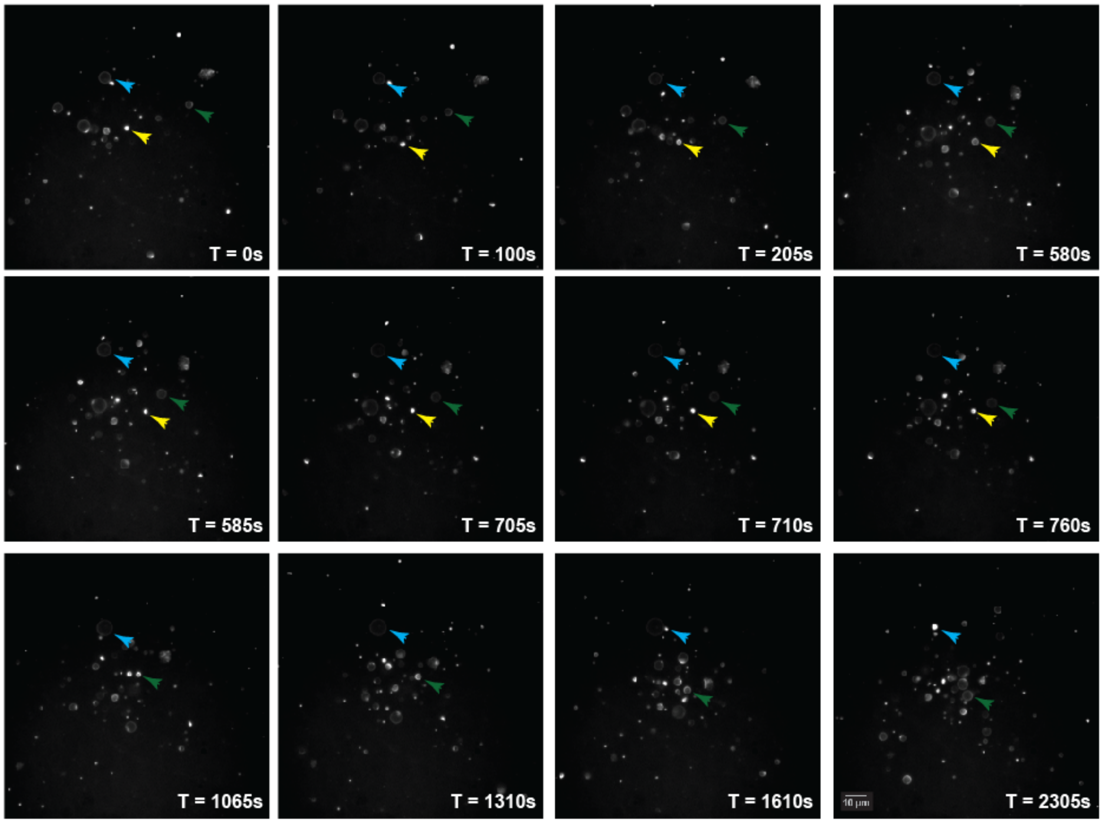

Figure S5: Time lapse of the phoenix behavior of the polymersomes. The green arrow highlights growing polymersomes and yellow and blue arrows point to polymersomes immediately after their collapse. The scale bar applies to all frames and corresponds to 10 µm. The data was collected at five second intervals. The sample stained with rhodamine 6G.

The phoenix behavior of the vesicles was observed by following individual vesicles through a micrograph time series as illustrated by the colored arrows in supporting Figure 5. The displayed micrographs were taken from a time series taken at five second intervals and lasting around three hours.

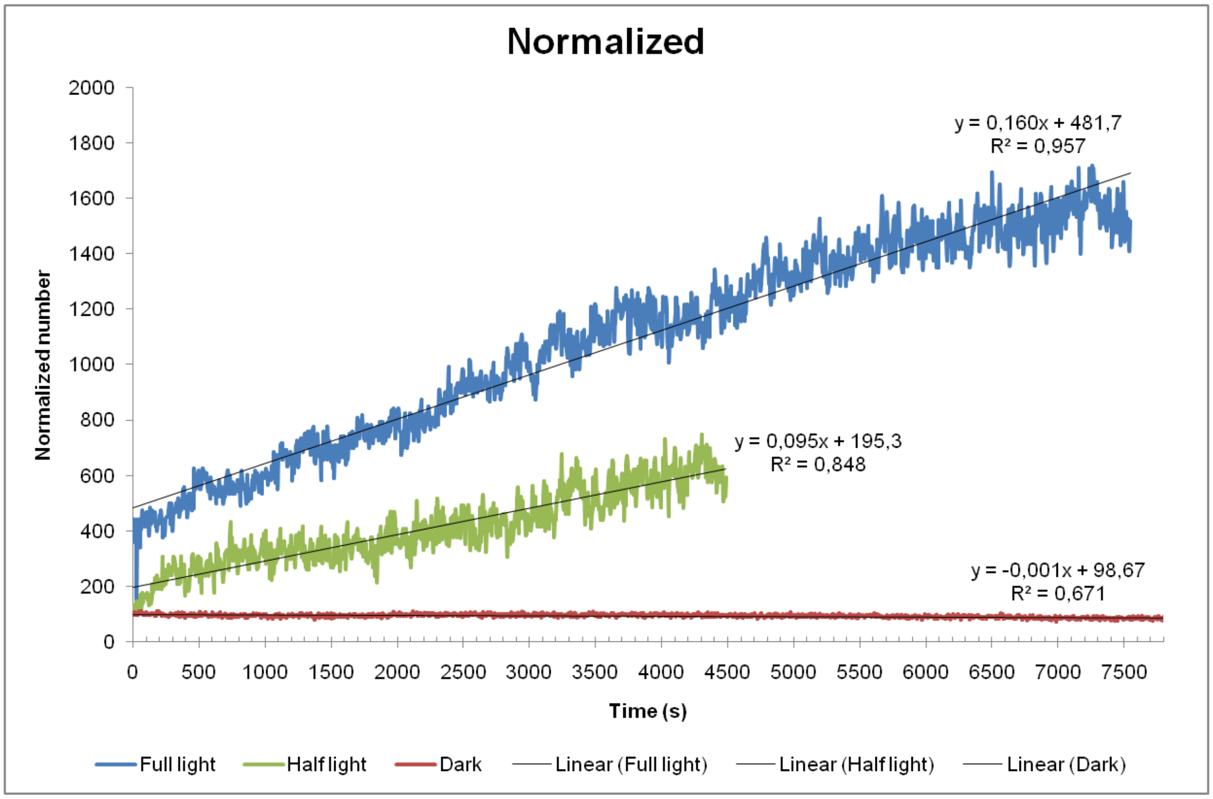

Figure S6: Influence of the light intensity on the PISA process shown as the normalized number of objects tracked normalized to an initial value of 100 at the onset of vesicle formation. Note the flat trend in the absence of light after the onset of vesiculation and the difference in the rate of formation between full and half-light during the investigation.

The evolution in the number of tracked aggregate objects in the solution after the onset of vesiculation depended on the amount of blue light the sample was exposed to, Supporting Figure 6. The slope clearly shows the correlation between the light exposure and the number of objects, as the light intensity doubles the slope increases by a factor of two. In the absence of light, the slope is roughly equal to zero. In order to simplify Supporting Figure, 6 the initial number of vesicles in the population was normalized to 100.

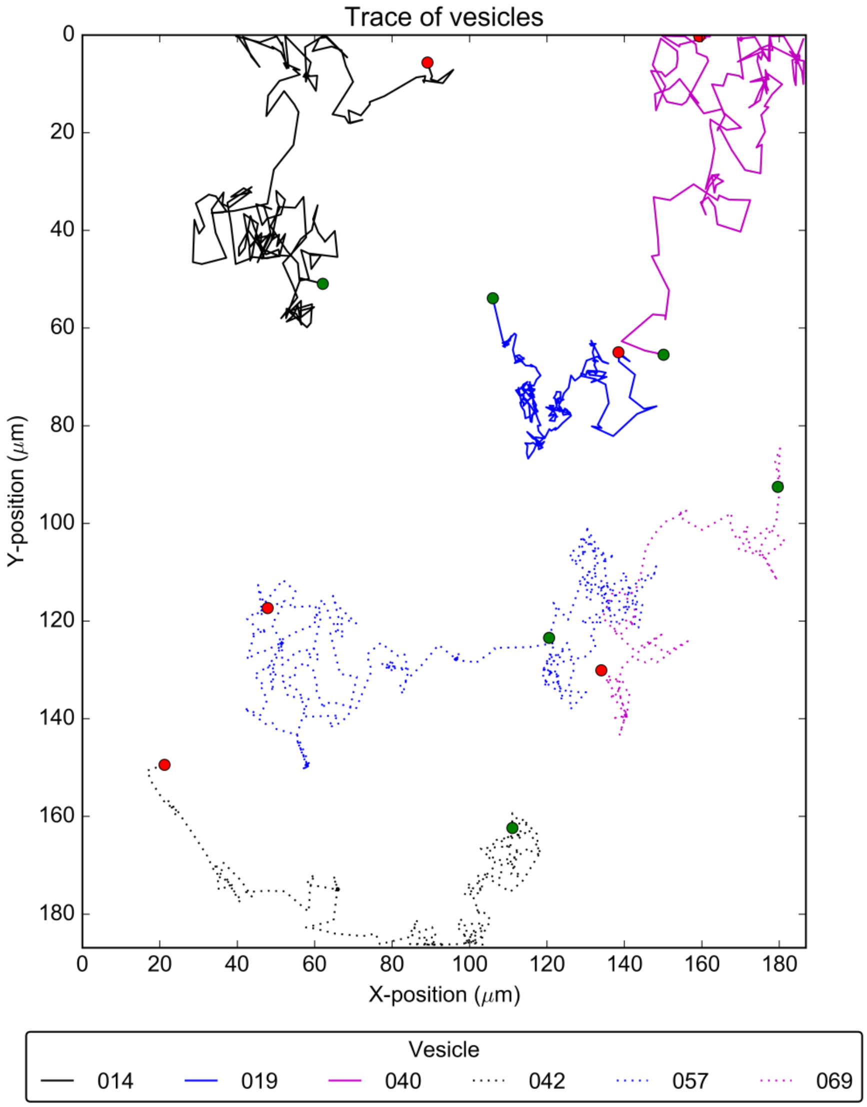

Figure S7: Trace of phoenix vesicle movement in the absence of blue light illumination. Note the absence of a clear collective direction of motion: the movement of the vesicles is in this instance similar to particle undergoing Brownian motion. The movement of the vesicles was monitored using rhodamine 6G and green light fluorescence microscopy.

In the absence of blue light illumination, the polymersomes did not move in any specific direction or towards a particular area, the computer traces of the vesicle motion are shown in Supporting Figure 7. In the dark the motion of the vesicles seemed to follow a random pattern, possibly originating from Brownian motion.

Movie 1.

This movie shows the dynamics of the PISA reaction mixture after vesicle formation. It details the emergent properties of the vesicles undergoing both "phoenix" cycles and phototaxis. The video covers 4520 seconds of blue light illumination (455-495 nm) with a frame recorded every 5 seconds using a green filter (excitation 540-590 nm, emission 600-650 nm, 50 ms exposure time) and Rhodamine 6G as the fluorophore.

References

1. Wan, W.-M. & Pan, C.-Y. One-pot synthesis of polymeric nanomaterials via RAFT dispersion polymerization induced self-assembly and re-organization. *Polym. Chem*. **1**, 1475-1484 (2010).
2. Sugihara, S., Blanazs, A., Armes, S. P., Ryan, A. J. & Lewis, A. L. Aqueous dispersion polymerization: a new paradigm for in situ block copolymer self-assembly in concentrated solution. *J. Am. Chem. Soc.* **133,** 15707-15713 (2011).
3. Zhang, X. et al. Well-defined amphiphilic block copolymers and nano-objects formed in situ via RAFT-mediated aqueous emulsion polymerization. *Macromolecules* **44**, 4149-4158 (2011).
4. Charleux, B., Delaittre G., Rieger, J. & D'Agosto F. Polymerization-induced self-assembly: from soluble macromolecules to block copolymer nano-objects in one step. *Macromolecules* **45**, 6753-6765 (2012).
5. Warren, N. J. & Armes, S. P. Polymerization-induced self-assembly of block copolymer nano-objects via RAFT aqueous dispersion polymerization. *J. Am. Chem. Soc*. **136**, 10174-10185 (2014).
6. Zehm, D., Ratcliffe, L. P. D. & Armes, S. P. Synthesis of diblock copolymer nanoparticles via RAFT alcoholic dispersion polymerization: effect of block copolymer composition, molecular weight, copolymer concentration, and solvent type on the final particle morphology. *Macromolecules* **46**, 128-139 (2013).
7. Fielding, L. A. *et al.*RAFT dispersion polymerization in non-polar solvents: facile production of block copolymer spheres, worms and vesicles in *n*-alkanes. *Chem. Sci.* **4**, 2081-2087 (2013).
8. Blanazs, A., Madsen, J., Battaglia, G., Ryan, A. J. & Armes, S. P. Mechanistic insights for block copolymer morphologies: how do worms form vesicles? *J. Am. Chem. Soc*. **133**, 16581-16587 (2011).
9. Yeow, J., Xu, J. & Boyer, C. Polymerization-induced self-assembly using visible light mediated photoinduced electron transfer-reversible addition-fragmentation chain transfer polymerization. *ACS Macro Lett.* **4**, 984-990 (2015).
10. Tan, J., Sun, H., Yu, M., Sumerlin, B. S. & Zhang, L. Photo-PISA: shedding light on polymerization-induced self-assembly. *ACS Macro Lett*. **4**, 1249-1253 (2015).
